# A Design Method of Styreneic Methyl Copolymers Normal Temperature Modified Asphalt Mixture Based on Performance Balance

**DOI:** 10.3390/ma15186193

**Published:** 2022-09-06

**Authors:** Limin Li, Zhaoyang Guo, Yuliang Lin

**Affiliations:** 1 School of Civil and Environmental Engineering, Hunan University of Science and Engineering, Yongzhou 425199, China; 2 Inner Mongolia Communications Construction Engineering Quality Supervision Bureau, Hohhot 010050, China; 3 Schools of Civil Engineering, Central South University, Changsha 410075, China

**Keywords:** design method, SMC, normal temperature, asphalt mixture, performance balance

## Abstract

The objective of this research was to develop a solution for the deterioration effect on the high-temperature rutting performance and water stability of SMC. This research proposed a method for designing an SMC normal temperature modified asphalt mixture based on the existing findings, experimental research and the performance balance. First, the power function curve model of the aggregate gradation was put forward. The 0.075 mm, 4.75 mm and nominal maximum particle size were the key points of the aggregate gradation, and their passing rate was about 6%, 30%, and 95% respectively. Then, on the basis of the quadratic curve model, a method for determination of the optimum asphalt aggregate ratio of SMC normal temperature modified asphalt mixture was put forward, considering the skeleton-density structure. Last, rutting tests, small beam bending tests, freeze-thaw split tests, permeability coefficient tests, texture depth tests and pavement roughness tests were conducted, and the test results all met the performance requirements of the specifications for the construction of highway asphalt pavement in China perfectly, especially the high-temperature and water stability properties, which indicated that the design method for SMC normal temperature modified asphalt mixtures based on performance balance presented in this paper was reasonable and practical.

## 1. Introduction

To enhance mixture sustainability, performance and workability, asphalt mixtures (AM) containing other additives such as binder modifiers, chemicals and rejuvenators have been widely used in recent years [1]. Styreneic methyl copolymers (SMC) normal temperature asphalt modifier is made up of methyl styrene block copolymer extracted from waste plastics, waste rubber and other additives in a certain proportion with the polymer solution [2]. The SMC modifier has good compatibility with the asphalt material, and it can greatly decrease the mixing temperature of the asphalt mixture [3]. It has the advantages of significant economic benefits, environmental protection, convenient construction and energy saving [4,5,6]. At present, many high- and low-grade highways are beginning to use SMC in China. Some studies on the SMC normal temperature modified asphalt mixture have been performed. The performance of the mixture under different SMC contents was studied by Luo et al. [7]. The results showed that the appropriate SMC content for asphalt concrete AC-13 and AC-20 was 6–10% and 6–8%, respectively. The performance of SMC recycled asphalt mixture at normal temperatures with a high reclaimed asphalt pavement (RAP) content was researched by some scholars [8,9,10,11,12]. The researchers found the application of an SMC normal temperature modifier could increase the amount of RAP. At the same time, the road performance of the SMC normal temperature modified mixture with high RAP could be ensured with appropriate SMC contents. Some researchers [13,14] have carried out research work on the performance of SMC normal temperature modified asphalt mixture. The results showed that with appropriate SMC contents, SMC normal temperature modifier could optimize low temperature crack resistance, but it had deterioration effect on high-temperature rutting performance and water stability.

The design method of the asphalt mixture is a main factor in asphalt pavement performance. In order to improve the scientificity of the design method of the asphalt mixture, numerous studies of mixture design methods have been performed. Wang et al. [15] studied recycled asphalt shingle modified asphalt mixture design and put forward a performance-engineered mix design approach which could provide mix designers with a reliable approach for designing innovative asphalt mixtures with a modern heterogeneous composition and higher recycling levels. Lv et al. [16] claimed that the mix design of the cold patching asphalt mixture (CPAM) should consider its own characteristics, and that the modified Marshall mix design method used as the mix design procedure for CPAM was feasible. Xin et al. [17] put forward a unique design method for the material composition of small particle-size (SPS) asphalt mixtures for controlling cracks in asphalt pavement. Xiao et al. [18] proposed a coarse aggregate void-filling (CAVF) method for designing the porous asphalt mixture, and found that it had advantages in designing an asphalt mixture with a skeleton interlocking structure. Fu et al. [19] put forward the design method of an asphalt mixture with fiber-reinforced performance based on the slip shear test of fiber asphalt mixture. Considering the application of foamed bitumen-stabilized RAP (recycled asphalt pavement) with certain gradation to base course, a design method of foamed bitumen mixture based on moisture susceptibility was put forward∙ by Shi et al. [20]. Zhang et al. [21] proposed the design method of emulsified asphalt mixture and claimed that it had good technical superiority. Liu [22] studied the design of mix proportions of SMC ultrathin overlays under normal temperatures. The results indicated that the optimum ratio of asphalt–aggregate of SMC-10 ultrathin overlays was 5.1%, and that all indicators of Marshall test and water stability could meet the technical specifications for the construction of highway asphalt pavement (JTG F40-2004) in China. The mix proportion design method and performance verification of SMC normal temperature recycled asphalt mixture was researched by Feng et al. [23]. The results showed that RAP content could be more than 60%, and that under the optimum asphalt aggregate ratio, the water stability performance and high-temperature performance results could meet the specifications. Based on the research on the properties of recycled asphalt mixtures with high RAP content and SMC at room temperature, a series method of grading design based on the closest state of the mineral material was obtained by Xie [24]. He found that the performance of high RAP-content SMC regenerated mixture at normal temperature could meet the requirements of technical index. In fact, because of the properties of asphalt mixtures influenced by the quantity of filler bitumen and asphalt mortar, the conventional asphalt mixture design method may not apply to all types of asphalt mixtures [25]. Studies on design methods for SMC normal temperature modified asphalt mixtures are very limited at present. The design of SMC normal temperature modified asphalt mixtures is still performed using the mix proportion design method of hot mix asphalt mixtures (Marshall mix design method).

Hence, in order to solve the problem of the deterioration effect on high-temperature rutting performance and water stability, it is necessary to investigate systematically the design method of SMC normal temperature modified asphalt mixture considering its performance balance on the basis of existing research and the characteristics of SMC.

## 2. Materials

In this research, Panjin 90# asphalt was chosen as the base asphalt sample, and SMC was provided by Ningxia Ruitai Tiancheng New Material Technology Co., Ltd., Yinchuan, China. The characteristic properties of Panjin 90# asphalt and SMC were given in Table 1 and Table 2, respectively. Based on existing findings [13], in the SMC normal temperature modified asphalt, the mass fraction of SMC was 12%. Crushed limestone was used as the coarse aggregate, fine aggregate and mineral filler. The test values in Table 3, Table 4 and Table 5 reflected the properties of the individual aggregate and mineral filler.

## 3. Design of the Aggregate Gradation

The skeleton structure of the coarse aggregate plays a very important role in the performance of asphalt mixtures, especially for enhancing the high-temperature properties of asphalt mixtures [28]. To research the skeleton structure of coarse aggregate, based on the existing research results [29,30,31,32], the grading curves of power function, exponential function and logarithmic function were adopted in this study. The power function, exponential function and logarithmic function were shown as the below formulae, respectively:

(1) $$y=a\cdot x^{b}$$

(2) $$y=c\cdot e^{dx}$$

(3) y=f⋅ln(x)+g

where a, b, c, d, f, g are regression coefficients, x is the particle size in mm, and y is the passing rate of the particle size in %.

**Figure 1:**
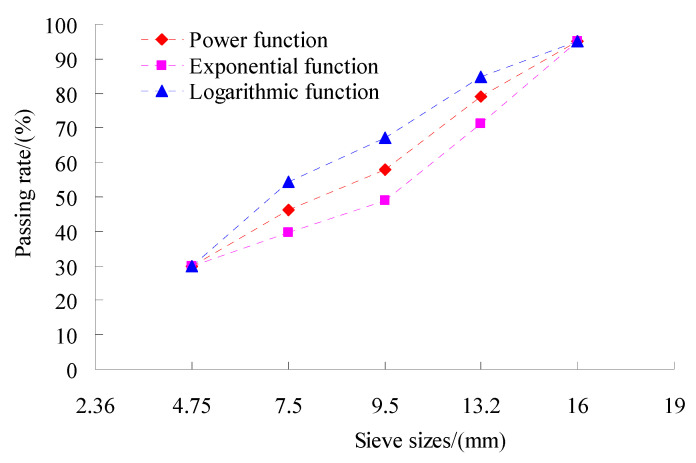
Three trial coarse aggregate gradations.

**Figure 2:**
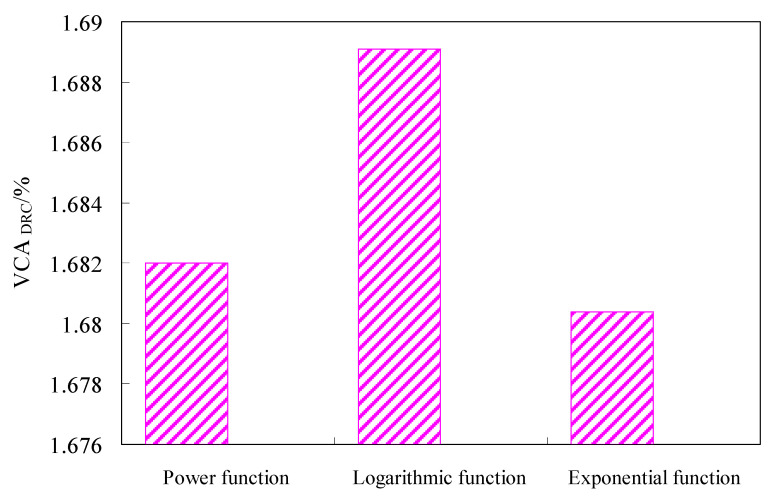
Tests results of bulk density and void ratio for three gradations.

To obtain the aggregate gradation, the control point and their passing rate were set up. Based on the previous research results [28,33], the 0.075 mm, 4.75 mm and nominal maximum particle size were chosen as the key points of the aggregate gradation. The nominal maximum diameter of coarse aggregates for the test was 16 mm in the study. The dividing size between coarse and fine aggregates was defined as 4.75 mm [28], and coarse and fine aggregates were calculated separately. The 0.075 mm and 4.75 mm passing rates were 6% and 30%, respectively, and the 16 mm passing rate was 95%. According to the power function, exponential function and logarithmic function, the passing rates of the differently sized aggregates were calculated, and the coarse aggregate grading curves obtained is shown in Figure 1. To study the skeleton structure of the coarse aggregate, the tests of their bulk density and void ratio were done according to test methods of aggregate for highway engineering (JTG E42-2005) in China. The test results of their percent voids in coarse mineral aggregate in the dry rodded condition (VCA_DRC_) were shown in Figure 2.

VCA_DRC_ can be used as the standard of criteria of skeleton formation of asphalt mixture [34]. It can be seen from Figure 2 that the VCA_DRC_ of the gradation of logarithmic function and exponential function were the largest and the smallest, respectively, and the VCA_DRC_ of the gradation of power function was in the middle of them. Considering the performance balance of the asphalt mixture, the gradation of power function should be selected at last.

**Figure 3:**
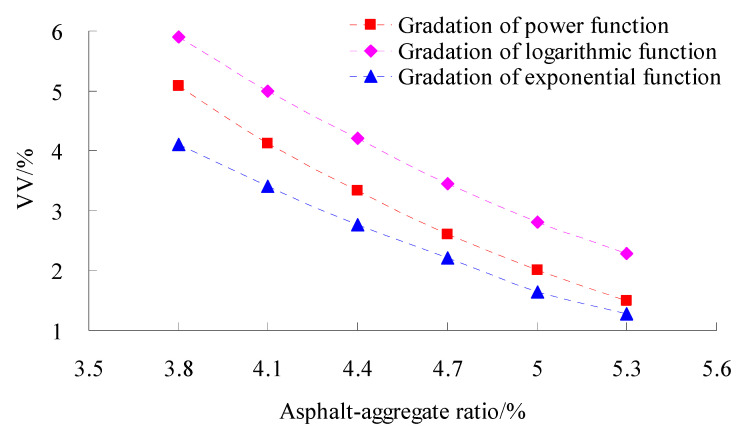
Tests results of VV.

**Figure 4:**
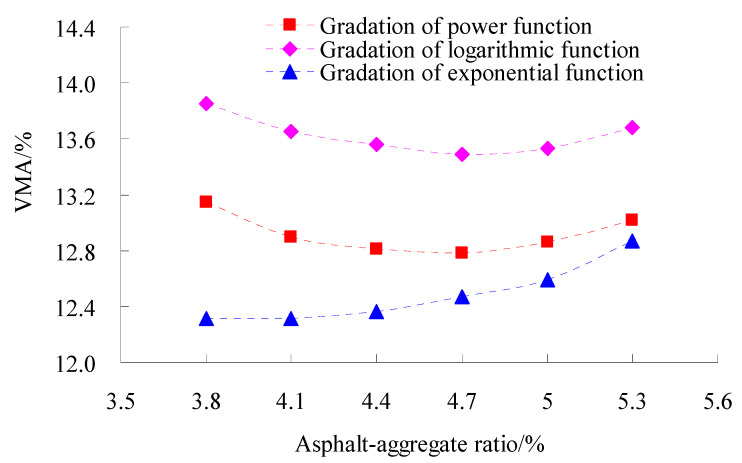
Tests results of VMA.

**Figure 5:**
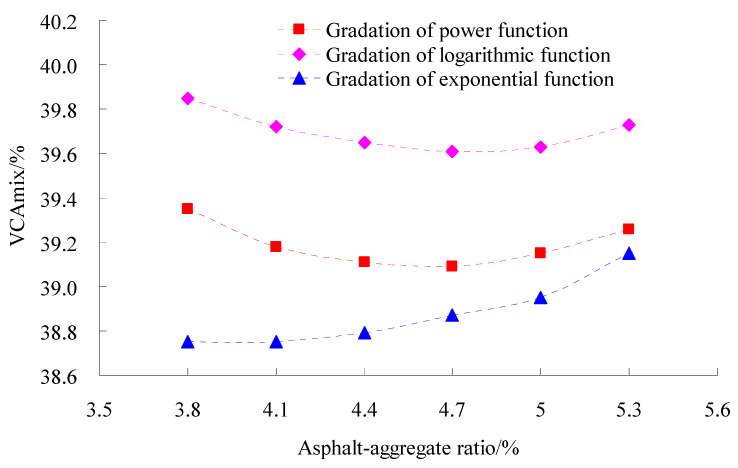
Tests results of VCAmix.

The volumetric parameters affect the performance of asphalt mixtures greatly [28]. In order to study the volumetric parameters of asphalt mixtures for different gradations, coarse aggregate gradation used the power function, exponential function and logarithmic function, respectively, and fine aggregate gradation adopted the power function. The tests of the Marshall Compaction test of three types of gradation were conducted according to standard test methods of bitumen and bituminous for highway engineering (JTG E20-2011) in China. The test results of the volume of air void (VV), voids in mineral aggregate (VMA) and percent voids in coarse mineral aggregate in bituminous mixtures (VCAmix) are shown in Figure 3, Figure 4 and Figure 5, respectively.

As observed in Figure 3, Figure 4 and Figure 5 at the same asphalt–aggregate ratio, the VV, VM and VCA_mix_ of the gradation of logarithmic function and exponential function were the largest and smallest, respectively, and the VV, VM and VCA_mix_ of the gradation of power function was in the middle of them.

**Figure 6:**
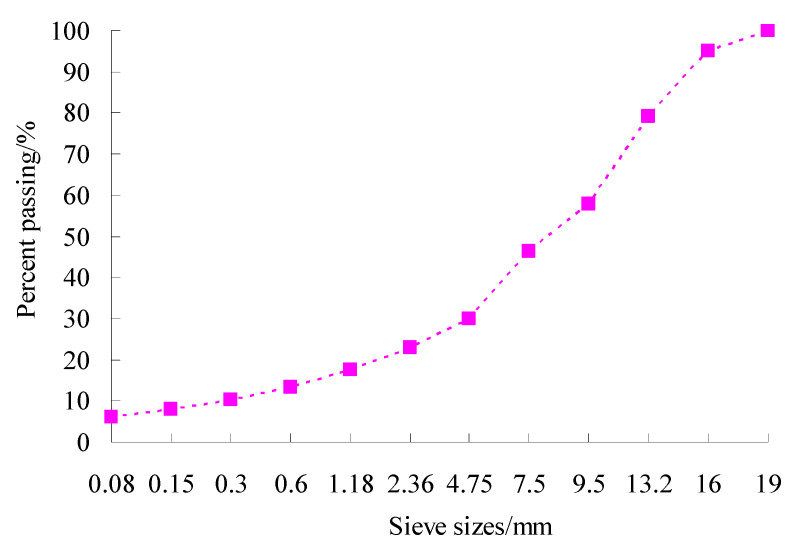
Trial aggregate gradation of power function.

In aggregate gradation, coarse aggregate performs a skeleton function, and its skeleton performance depends on the anti-rutting ability of the asphalt mixture [31]. The appropriate value of volumetric parameters is a key factor to ensure skeleton structure formation in asphalt mixtures [28]. The test results of bulk density, void ratio and their Marshall compaction test indicated that regarding skeleton-density structure as the target, an asphalt mixture of gradation of power function was reasonable. Considering that SMC normal temperature modifier had a deterioration effect on the high-temperature rutting performance-based performance balance of asphalt mixture, the gradation design model of power function was adopted at last, and the test gradation used is given in Figure 6.

## 4. Determination of Optimum Asphalt Aggregate Ratio

The asphalt–aggregate ratio has a significant influence on the performance of the mixture and should be strictly controlled within a reasonable range. It is very important for the comprehensive performance of asphalt mixture to keep a good skeleton-density structure under the adopted optimum asphalt–aggregate ratio. The material properties of the asphalt mixture are similar to those of inorganic binding material. Therefore, it is feasible to adopt the method for determining optimum water content using in the inorganic binding material to determine the optimum asphalt–aggregate ratio of the asphalt mixture. Assuming that the asphalt is the water in the inorganic binding material, the asphalt–aggregate ratio is considered as the optimum water content. The $\rho_{d}$ can be used to evaluate the most compact state of asphalt mixture, and it can be calculated as in the formula below [35].

(4) $$\rho_{d}=\rho_{f}\times \frac{100}{100+p_{a}}$$

where $\rho_{d}$ and $\rho_{f}$ are the dry density of the asphalt mixture and the bulk density of the bituminous mixtures, respectively, and $p_{a}$ is the optimum bitumen–aggregate ratio of the asphalt mixture.

In fact, the VMA and VCA_mix_ can be used to evaluate the most compact state of the asphalt mixture, too, and their calculation formulas are as follows [35].

(5) $$\mathrm{VMA}=1-\frac{\rho_{f}}{\gamma_{sb}}\times \frac{100}{100+p_{a}}$$

where $\gamma_{sb}$ is the mineral aggregate gross volume density of asphalt mixture.

(6) $$VCA_{mix}=1-\frac{\rho_{f}}{\gamma_{sb,ca}}\times \frac{100}{100+p_{a}}\times P_{ca}^{\prime }$$

where $\gamma_{sb,ca}$ is the bulk density of coarse aggregate, and $P_{ca}^{\prime }$ is the mass percentage of coarse aggregate in the mineral aggregate.

The quadratic curve model can be used to analyze the relationship between the asphalt–aggregate ratio and $\rho_{d}$, VMA and VCA_mix_ [35,36]. On the basis of their maximum or minimum value of the quadratic curve model, their optimum asphalt aggregate ratio can be obtained, respectively. Theoretically, the optimum asphalt–aggregate ratio of the mixture should be completely equal based on the maximum or minimum value of $\rho_{d}$, VMA and VCA_mix_. However, due to test error, there may be some error in their optimum asphalt–aggregate ratio. Therefore, the optimal asphalt–aggregate ratio of the asphalt mixture is calculated according to Formula (7):

(7) $$AM_{opt}=average\left(AM_{\rho },AM_{VCA},AM_{VMA}\right)$$

where $AM_{opt}$ is the optimal asphalt–aggregate ratio of the asphalt mixture and $AM_{\rho }$, $AM_{VCA}$, $AM_{VMA}$ are the optimum asphalt–aggregate ratio of the mixture based on the maximum or minimum values of $\rho_{d}$, VMA and VCA_mix_, respectively.

**Figure 7:**
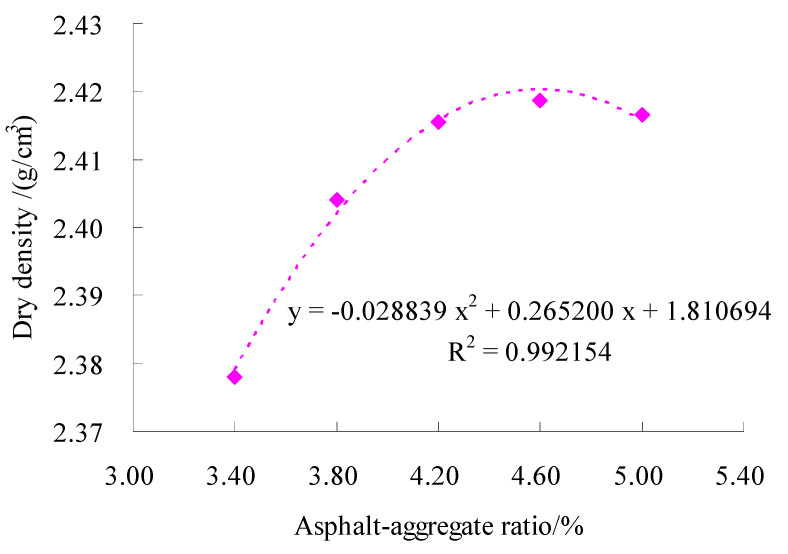
Test results of dry density at different asphalt–aggregate ratios.

**Figure 8:**
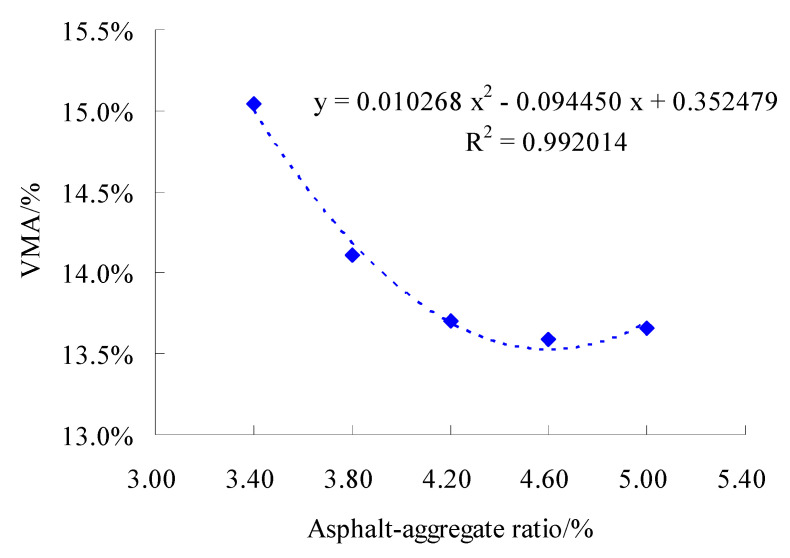
Test results of VMA at different asphalt–aggregate ratios.

**Figure 9:**
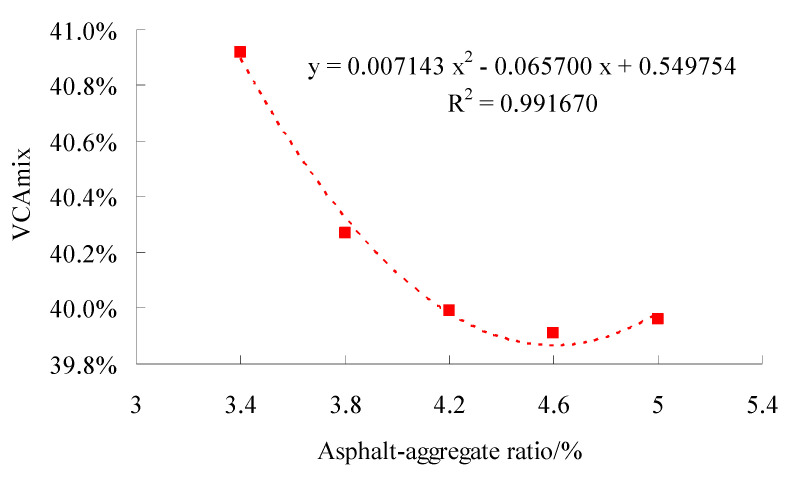
Test results of VCA_mix_ at different asphalt—aggregate ratios.

The gyratory compaction method was used to form the specimen with both sides 100 times. For test gradation, the Marshall compaction test of the SMC normal temperature modified asphalt mixture were conducted according to the standard test methods of bitumen and bituminous for highway engineering (JTG E20-2011) in China. The calculation results of the Marshall test of the finished formed specimens are shown in Figure 7, Figure 8 and Figure 9, respectively.

According to Figure 7, Figure 8 and Figure 9, the regression equation of three volumetric parameters and asphalt–aggregate ratio can be obtained. For $\rho_{d}$, there was

(8) $$y=-0.028839x^{2}+0.265200x+1.810694$$

For VMA, there was

(9) $$y=0.010268x^{2}-0.094450x+0.352479$$

For VCA_mix_, there was

(10) $$y=0.007143x^{2}-0.065700x+0.549754$$

According to Formulas (8)–(10), we obtained $AM_{\rho }=4.5979\%$, $AM_{VMA}=4.5992\%$ and $AM_{VCA}=4.5989\%$. Then, based on Formula (7), there was $AM_{opt}=4.6\%$.

## 5. Performance Verification

On the basis of optimum asphalt–aggregate ratio, the performance tests of SMC normal temperature modified asphalt mixture of the trial aggregate gradation were conducted. The tests were performed according to the standard test methods for bitumen and bituminous mixtures for highway engineering (JTG E20-2011) in China.

### 5.1. Anti-Rutting Performance

**Figure 10:**
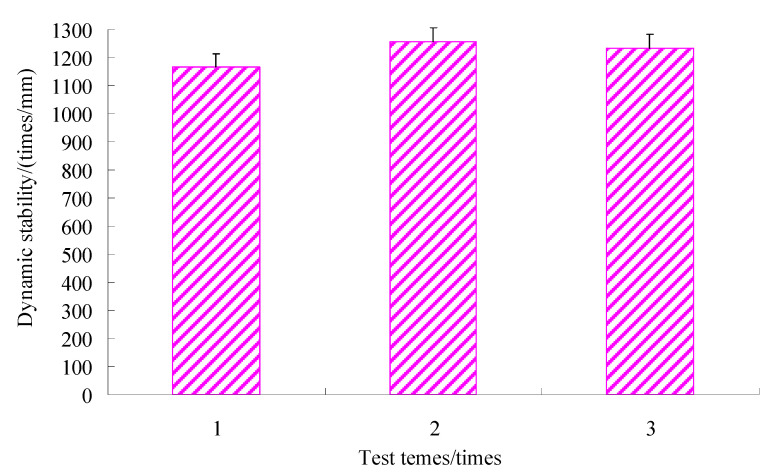
Dynamic stability of the rutting test at 60 °C.

Rutting tests were conducted to estimate the rutting resistance of the asphalt mixtures using a rutting tester from China. The rutting depth for the initial stage was measured under testing with the wheel-pressure of 0.7 MPa and a temperature of 60 °C. The results are shown in Figure 10.

It can be seen from Figure 10 that the dynamic stability of the SMC normal temperature modified asphalt mixture of trial aggregate gradation with 4.6% asphalt content was more than 800 times/mm, which meets the hot summer area requirements of the specifications (JTG F40-2004) for the construction of highway asphalt pavement in China.

### 5.2. Water Stability

The water stability of the asphalt mixture is usually evaluated using the freeze–thaw split test at 25 °C, with a loading rate of 50 mm/min [37]. The freeze–thaw splitting tests were carried out according to the Freeze–Thaw Split Test of Asphalt Mixture (T0729-2000). The freeze–thaw splitting strength ratio is the ratio of the freeze–thaw splitting strength to splitting strength. A higher splitting strength ratio corresponds to a higher water stability. The results are listed in Table 6.

Table 6 showed that SMC normal temperature modified asphalt mixture could meet the requirements of the freeze–thaw splitting strength ratio, which was more than 80%.

### 5.3. Low Temperature Anti-Cracking Performance

Small beam bending tests were conducted using an UTM-100 material testing system at −10 °C, with a loading rate of 50 mm/min. The size of the trabecular is 250 mm × 30 mm × 35 mm. For the small beam bending test, the fracture strain is the important evaluation index. A larger value of fracture strain indicates a better low-temperature anti-cracking performance for the SMC normal temperature modified asphalt mixture. The results were as shown in Table 7.

Table 7 showed that the strain failure value at low temperatures was more than 3000 με. According to the technical specifications for the construction of highway asphalt pavement (JTG F40-2004) in China, the SMC normal temperature modified asphalt mixture can meet the requirements for zones with a minimum temperature of less than −37 °C.

### 5.4. Engineering Application

**Figure 11:**
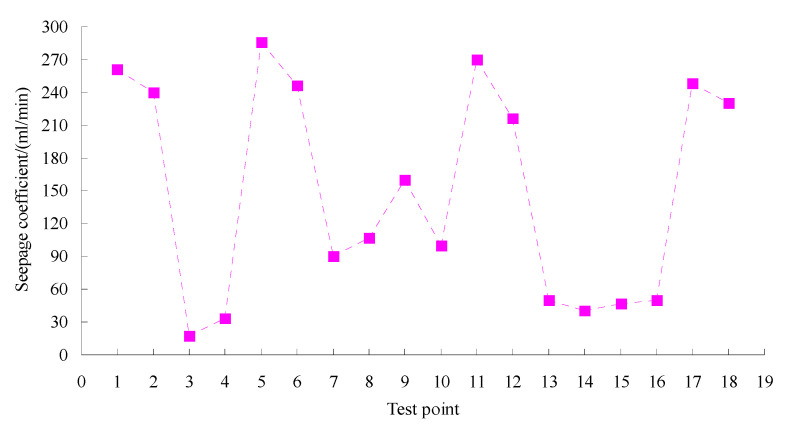
Test results of permeability coefficient for test road.

**Figure 12:**
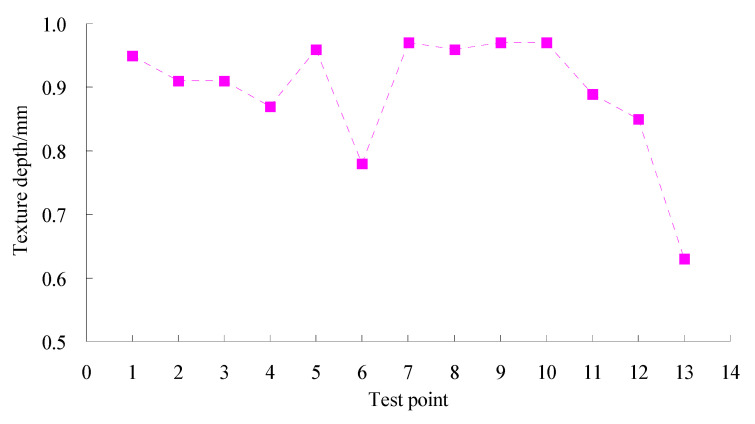
Test results of texture depth for test road.

**Figure 13:**
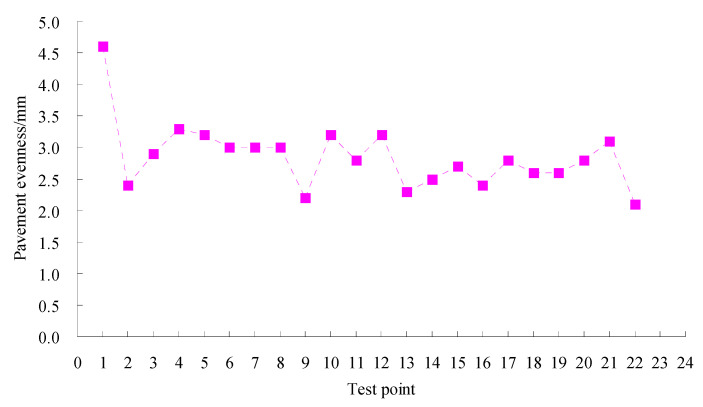
Test results of pavement roughness for test road.

The SMC normal temperature modified asphalt mixture has been used in highway construction in Xinjiang in China. The tracking test was done according to field test methods for highway subgrade and pavement (JTG 3450-2019) in China. Permeability coefficient tests were conducted using an HHDS-2/3 pavement seepage meter made by Cangzhou Zerui Test Instrument Co., Ltd., China according to the test methods for the permeability coefficient of asphalt pavement (T0971-2019). The permeability coefficient results of the test road in two years are shown in Figure 11. Texture depth tests were performed using an LD-138 electric sand paver made by Hebei Besta Test Instrument Co., Ltd., China based on the method of testing pavement structure depth with electric sand paver (T0962-1995). The texture depth results of the test road in two years are given in Figure 12. Pavement roughness tests were done using a JZCG2 three-meter ruler made by Hangzhou PUEN Technology Co., Ltd., China on the basis of the testing method of flatness with three-meter ruler (T0931-2008). The test results of the pavement roughness of the test road in two years are shown in Figure 13.

The gradation composition of an asphalt mixture has an indirect effect on the asphalt mixture’s permeability coefficient, and the permeability coefficient can indicate the water stability of asphalt pavement. The permeability coefficient of asphalt is smaller, and its water permeability is better. It can be seen from Figure 11 that their test values were all less than 300 mL/min, which meets the quality inspection and evaluation standards for highway engineering (TG F80/1-2004) [38]. The results indicated that the gradation composition of asphalt mixture was reasonable, and that the asphalt pavement possessed a fine water-resisting property and was enabled to meet service demands.

Texture depth can evaluate the drainage performance and skid resistance of asphalt pavement. The gradation has an important effect on texture depth. As observed in Figure 12, the test values of texture depth were between 0.5 and 1 mm, which meets the quality inspection and evaluation standards for highway engineering (JTGF80/1-2004). The results indicated that the gradation composition of the asphalt mixture was reasonable, and that the asphalt pavement possessed a fine drainage performance and skid resistance and was enabled to meet service demands.

Pavement roughness is a main index used to evaluate the service performance of highway pavement. A smaller pavement roughness corresponds to a better service ability. It can be seen from Figure 13 that the test values were all less than 5 mm, which meets the quality inspection and evaluation standards for highway engineering (TG F80/1-2004). The results indicated that the asphalt pavement possessed a fine rutting resistance and was enabled to meet service demands.

## 6. Conclusions

The SMC normal temperature modified asphalt mixture has the advantages of significant economic benefits, environmental protection, convenient construction and energy saving, but it had a deterioration effect on high-temperature rutting performance and water stability. Based on the existing findings and experimental research, a design method for SMC normal temperature modified asphalt mixture based on performance balance was proposed. A series of performance tests of SMC normal temperature modified asphalt mixtures designed using the design method based on performance balance were carried out. The tests included rutting tests, small beam bending tests, freeze–thaw split tests, permeability coefficient tests, texture depth tests and pavement roughness tests. The results of the tests verified the usefulness and feasibility of the design method based on performance balance. Based on the results of this limited laboratory investigation, the following conclusions can be drawn.

(1) Considering that the skeleton structure could enhance the high temperature of SMC normal temperature modified asphalt mixture, a recommended method for designing the gradation of SMC normal temperature modified asphalt was the power function curve model of the aggregate gradation. The key points of the aggregate gradation of 0.075 mm, 4.75 mm and the nominal maximum particle size were adopted in the method. The passing rate of 0.075 mm and 4.75 mm should be restricted at 6% and 30%, respectively, and the passing rate of the nominal maximum particle size should be kept at about 95%.
(2) Considering the skeleton-density structure could solve the performance balance of SMC normal temperature modified asphalt mixture especially in terms of enhancing its high-temperature and water stability properties, on the basis of the quadratic curve model, a method for the determination of optimum asphalt aggregate ratio of SMC normal temperature modified asphalt mixture was put forward according to the relationship between asphalt aggregate ratio and $\rho_{d}$, VMA and VCA_mix_.
(3) The performance of SMC normal temperature modified asphalt mixture designed by using the design method based on performance balance could meet the requirements of the technical specifications perfectly. The method was effective in improving the overall performance of SMC normal temperature modified asphalt mixture. It is expected that more engineering project verifications will be conducted in future studies.

## Figures and Tables

**Table 1:** Properties of base asphalt.

Properties		Criteria	Results of Panjin 90#	Methods
Ductility at 15 °C (cm)		≥100	>100	T0605-2011 [26]
Penetration degree at 25 °C (0.1 mm)		80~100	83	T0604-2011 [26]
Softening point (°C)		≥42	49.5	T0606-2011 [26]
Flash point (°C)		≥245	285	T0611-2011 [26]
Wax content		≤2.2	1.8	T0615-2011 [26]
Penetration index		−1.5~+1.0	−0.4	T0604-2011 [26]
After TFOT (163 °C, 5 h)	Mass loss (%)	±0.8	0.035	T0609-2011 [26]
	Penetration degree ratio at 25 °C (%)	≥57	65	T0604-2011 [26]
	Ductility at 15 °C (cm)	≥8	14	T0605-2011 [26]

**Table 2:** SMC normal temperature asphalt modifier properties.

Properties	Colour	Density (g/cm^3^)	Rubber Hydrocarbon Content (%)	Rotation Viscosity at 25 °C	Flash Point	Benzene Content of Volatile Organic Compounds
				(Pa⋅s)	(°C)	
Criteria	-	0.8~1.0	>85	≤0.8	90~110	≤0.1
Results	Blown	0.88	83	0.71	66	0.07

**Table 3:** Coarse aggregate properties.

`Technical Indexes	Criteria	Results	Methods
Apparent density (g/cm^3^)	≥2.5	2.704	T0304-2005 [27]
Crush value (%)	≤28	18.3	T0316-2005 [27]
Content of acicular and flaky shape particles (%)	≤20	10.1	T0304-2005 [27]
Losses of Los Angeles Abrasion Test (%)	≤30	17.2	T0316-2005 [27]
Water absorption (%)	≤3	0.701	T0304-2005 [27]
Asphalt adhesion (graduation)	≥4	4	T0616-1993 [26]
Firmness (%)	≤12	9	T0314-2005 [27]
Mud content (%)	≤3	0.1	T0320-2005 [27]
Impact value (%)	≤28	14.3	T0322-2000 [27]

**Table 4:** Fine aggregate properties.

Properties	Criteria	Results	Methods
Apparent density (g/cm^3^)	≥2.50	2.617	T0328-2005 [27]
Water absorption (%)	≤2	1.82	T0340-2005 [27]
Sand equivalent (%)	≥50	53	T0334-2005 [27]

**Table 5:** Mineral filler properties.

Properties		Criteria	Results	Methods
Apparent density(g/cm^3^)		≥2.50	2.661	T0352-2005 [27]
Water content (%)		≤1	0.21	T0305-1994 [27]
Hydrophilic coefficient		<1	0.6	T0353-2005 [27]
Size distributions (%)	<0.6 mm	100	100	T0351-2005 [27]
	<0.15 mm	90~100	99.3	
	<0.075 mm	75~100	85.7	

**Table 6:** Results of the freeze–thaw split test.

Freeze-Thaw Splitting Strength (MPa)	Splitting Strength (MPa)	Freeze-Thaw Splitting Strength Ratio (%)	Criteria
0.53	0.60	87.3	≥80%

**Table 7:** Results of the small beam bending test at −10 °C.

Flexural Tensile Strength (MPa)	Failure Strain (με)	Failure Stiffness Modulus (MPa)
3.96	3064.27	1292.31

## References

[1] Cadorin N.D., Melo J., BroeringW B., Manfro A.L., Barra B.S. (2021). Asphalt nanocomposite with titanium dioxide: Mechanical, rheological and photoactivity performance. Constr. Build. Mater..

[2] Sun Y., He D. (2021). High and Low-Temperature Performance Evaluation and Microanalysis of SMCSBS Compound-Modified Asphalt. Materials.

[3] Tan Y., Zhang H., Cao D., Xia L., Du R., Shi Z., Dong R., Wang X. (2019). Study on cohesion and adhesion of high-viscosity modified asphalt. Int. J. Tran. Sci. Tech..

[4] Tan Y., Zhang L., Xu H. (2012). Evaluation of low-temperature performance of asphalt paving mixtures. Cold. Reg. Sci. Technol..

[5] Zhao L. (2017). Application of SMC modified asphalt at room temperature on G312 Line Manag. Tech. SME.

[6] Qu H. (2016). Application of SMC modified asphalt at room temperature in highway overlay engineering. Shanxi Constr..

[7] Luo H., Li Z., Zheng P., Ouyang C., Qiu Y. (2020). Analysis of road performance, mechanism and environmental protection benefit of SMC room temperature modifier. J. Build. Mater..

[8] Liang Q. (2020). Research on the performance of high RAP content SMC room *temperature* recycled asphalt mixture. West. Commun. Tech..

[9] Shu P., Wang B., Zhang Y., Wang S. (2020). Research on performance of high RAP content SMC normal temperature recycled asphalt mixture in Panzhihua area. Transp. Energy Conserv. Environ. Prot..

[10] Tian M., Li Y., Luo D., Wang Z. (2020). Study on fatigue performance of SMC recycled modified asphalt mixture with 50% RAP. J. Highw. Transp. Res. Dev. (Appl. Technol. Ed.).

[11] Tian M., Feng Z., Ya F., Yan J. (2018). The SMC normal temperature reclaimed asphalt mixture mix design and performance evaluation research. DEStech Trans. Eng. Technol. Res..

[12] Liu X., Xu G., Liu J., Wang S. (2020). Study on the optimum content of recycling agent for SMC normal temperature recycled asphalt mixture on S214 line of Panzhihua. Transp. Energy Conserv. Environ. Prot..

[13] OuYang Y. (2017). The Evaluation of Comprehensive Performance of SMC Normal Temperature Modified Asphalt and the Asphalt Mixture. Master’s Thesis.

[14] Zhu F., Ma Q., Li Y., Hui J., Xiao Y., Chen Y. (2021). Construction of SMC normal temperature asphalt mixture in Dongning area technical study. TranspoWorld.

[15] Wang H., Rath P., Buttlar W.G. (2020). Recycled asphalt shingle modified asphalt mixture design and performance evaluation. J. Traffic Trans. Eng. (Engl. Ed.).

[16] Lv S., Wang S., Xia C., Liu C. (2020). A new method of mix design for cold patching asphalt mixture. Front. Mater..

[17] Xin J., Pei J., Akiyama M., Li R., Zhang J., Shao L. (2019). A study on the design method for the material composition of small particle-size asphalt mixture for controlling cracks in asphalt pavement. Appl. Sci..

[18] Xiao X., Zhang X., Xiao X. (2016). Design of porous asphalt mixture based on CAVF method. J. Highw. Transp. Res. Dev..

[19] Fu Z., Wang B., Li J., Sun Q., Zhang P. (2018). Design method for fiber asphalt mixture based on fiber reinforcement performance. Road Mach. Constr. Mech..

[20] Shi F., He Z., LV W., Xu B., Zhu L. (2004). Design method and laboratory research of foamed bitumen mixture. J. Highw. Transp. Res. Dev..

[21] Zhang Y., Liu Z. (2016). Study on the design method of emulsified asphalt mixture. Highw. Eng..

[22] Liu J. (2018). Design of mix proportion and performance validation of SMC ultrathin overlays under normal temperature. Railw. Conctruction Technol..

[23] Feng Z., Li Y., Wang S. (2018). Mix proportion design method and performance verification of SMC normal temperature recycled asphalt mixture. J. Highw. Transp. Res. Dev. (Appl. Technol. Ed.).

[24] Xie Z. (2017). Research on Properties of Recycled Asphalt Mixture with High RAP Content and SMC at Room Temperature. Master’s Thesis.

[25] Ozawa K. (2002). Design Method for mixing formulas of asphalt mixtures considering aggregate voids. J. Jpn. Pet. Inst..

[26] China Ministry of Transport (2011). JTG E20-2011 Specifications and Test Methods of Bitumen and Bituminous Mixtures. For Highway Engineering.

[27] Ministry of Communications of the PRC (2005). JTGE42-2005Test Methods of Aggregate for Highway Engineering.

[28] Zhao Y., Xu T., Huang X., Li Z. (2012). Gradation design of the aggregate skeleton in asphalt mixture. J. Test. Eval..

[29] Zhang X., Guo Z., Wu K. (1995). Volume method of bituminous mixture design. J. Harbin Univ. Archit.Eng..

[30] Vavrik W., Pine W., Carpenter S. (2002). Aggregate blending for asphalt mix design bailey method. Transp. Res. Rec. J. Transp. Res. Board.

[31] Zhang Y., Zheng M., Hu G., Zhu Q. (2013). Coarse aggregate gradation design index and method of asphalt mixture. Adv. Mater. Res..

[32] Xu S., Peng G., Zhang Y., Guo Y. (2021). Design Method of asphalt pavement mixture based on performance balance approach. J. Transp. Eng. Part B Pavements.

[33] Li L. (2011). Design method study on the large-stone flexible base durable asphalt pavement of resisting rutting. Ph.D. Thesis.

[34] Miao C., You H., Lu Y., Zuo G. (2009). Analysis and discuss on the inspection method of skeleton framework formation of bituminous mixture. J. Transp. Eng. Inf..

[35] Huang Y., Liu C., Wang X., Yang G. (2016). Proportion design method of asphalt mixture based on the most compact state of skeleton. J. China Foreign Highw..

[36] Liu S., Luan J., Xue Z., Chen L., Cao W. (2022). Model and analysis of voids in the mineral aggregate for the same type of hot mix asphalt at different gradations. J. Build. Mater..

[37] Li L., Guo Z., Ran L., Zhang J. (2020). Study on low-temperature cracking performance of asphalt under heat and light together conditions. Materials.

[38] Ministry of Communications of the PRC (2004). JTGF80/1-2004 Quality Inspection and Evaluation Standards for Highway Engineering.

